# Case report: Renal malformations in wild roe deer (*Capreolus capreolus*) in Central Poland

**DOI:** 10.3389/fvets.2025.1523216

**Published:** 2025-02-18

**Authors:** Katarzyna Kliczkowska, Wojciech Bielecki, Marta Kloch, Marcin Świątek, Daniel Klich

**Affiliations:** ^1^Department of Pathology and Veterinary Diagnostics, Institute of Veterinary Medicine, Warsaw University of Life Sciences (SGGW), Warsaw, Poland; ^2^Department of Animal Genetics and Conservation, Institute of Animal Sciences, Warsaw University of Life Sciences (SGGW), Warsaw, Poland; ^3^Department of Animal Breeding and Nutrition, Institute of Animal Sciences, Warsaw University of Life Sciences (SGGW), Warsaw, Poland

**Keywords:** roe deer, renal hypoplasia, nephritis, malformation, Poland

## Abstract

Kidney diseases are observed in various wild animal species, more often noted in captive than in free-ranging animals. There are few reports in roe deer (*Capreolus capreolus*). Two kidney malformations were encountered while reviewing and collecting the roe deer samples. Kidney malformations were found in samples from two roe deer in Central Poland, one in 5-year-old female near Węgrów (50 km northeast of Warsaw) and one in 7-year-old male near Rawa Mazowiecka (75 km southwest of Warsaw). The female had a smaller (5 cm long and weighed 20 g) shrunken kidney (no prominent histopathological changes) and compensatory hypertrophy of the other kidney (weighting 85.8 g). A diagnosis of renal hypoplasia was made. The male had a smaller kidney (4.5 cm long and weighed 15.3 g) and normal-sized the other kidney (7.5 cm and 55.6 g). Massive mononuclear infiltrates composed mainly of lymphocytes and plasma cells in the renal interstitium, with hyperplasia of the connective tissue and multifocal glomerular sclerosis, were found in both kidneys. Diagnosis of chronic interstitial nephritis was made.

## 1 Introduction

Kidney diseases are observed in various wild animal species (1–4). However, these diseases are much more often observed in captive than in free-ranging animals, which is a natural result of the possibility of observing and thoroughly examining them. Some kidney diseases can result in bilateral or unilateral kidney malformations, which can be easily detected during initial examination on the spot (5). These diseases include developmental disorders such as hypoplasia and dysplasia, as well as changes resulting from chronic interstitial nephritis. Few cases of these changes in wild deer are described in the literature. Among them, developmental disorders, primarily polycystic kidney disease (PKD) was noted in roe deer (*Capreolus capreolus*) (6, 7) or white-tailed deer (*Odocoileus virginianus*) (8). In other studies, chronic interstitial nephritis associated with listeriosis has been described in red deer (*Cervus elaphus*) (9), and similar histopathological changes were also described in this species (10). One case of renal hypoplasia without a detailed description was reported in a roe deer in Switzerland as an additional finding (7).

Detecting diseases in wildlife is a major challenge and requires organized monitoring, usually based on collecting postmortem samples (11–13). For this reason, it is crucial to report cases, especially about poorly known diseases. Such monitoring based on collecting postmortem samples from roe deer was conducted in Poland. While reviewing and collecting the material, two cases of kidney malformations were encountered, and this report aims to present these case studies, thereby contributing to the understanding of renal malformations in wild roe deer.

During the project, roe deer dissections were performed by designated hunters, according to standard dissection techniques, using the Polish protocol for animal infectious diseases (14). In total, we collected organs from 173 roe deer, of which 167 with at least one kidney. Organs were collected into plastic bags. In case of any lesions, fragments of organs were collected into 10% buffered formalin from the margin of the lesion, collecting both affected and normal tissue. Organs, if possible, were weighed, measured, and subjected to further analysis depending on noted macroscopic lesions or malformations. Kidney malformations were found in samples from two roe deer in Central Poland. Both animals ranged in the area of a large wind farm.

For histopathological examination, representative samples were fixed in 10% buffered formalin, embedded in paraffin wax, cut in 4 μm sections, and stained with hematoxylin and eosin (H&E). Additional slides were stained with Masson’s Trichrome stain for collagen and Koss stain for calcium. Histopathological slides were evaluated with light microscopy using LEICA DM 1000 microscope.

Due to histopathological changes, material from male roe deer (Case 2) was tested for *Leptospira* spp. Tissue fluid samples were used to detect Ig- and IgM-specific Leptospira antibodies by VetLine Leptospira IgM ELISA and VetLine Leptospira IgG ELISA kits (NovaTec Immundiagnostica GmbH, Dietzenbach, Germany). Both ELISA were performed according to the manufacturer instructions. The absorbance of each well was read at a wavelength of 450 nm by EPOCH spectrophotometer (BioTek Instruments Inc., Winooski, VT, USA). Results were interpreted based on the cut-off values given by the manufacturer.

## 2 Case 1

**Figure 1:**
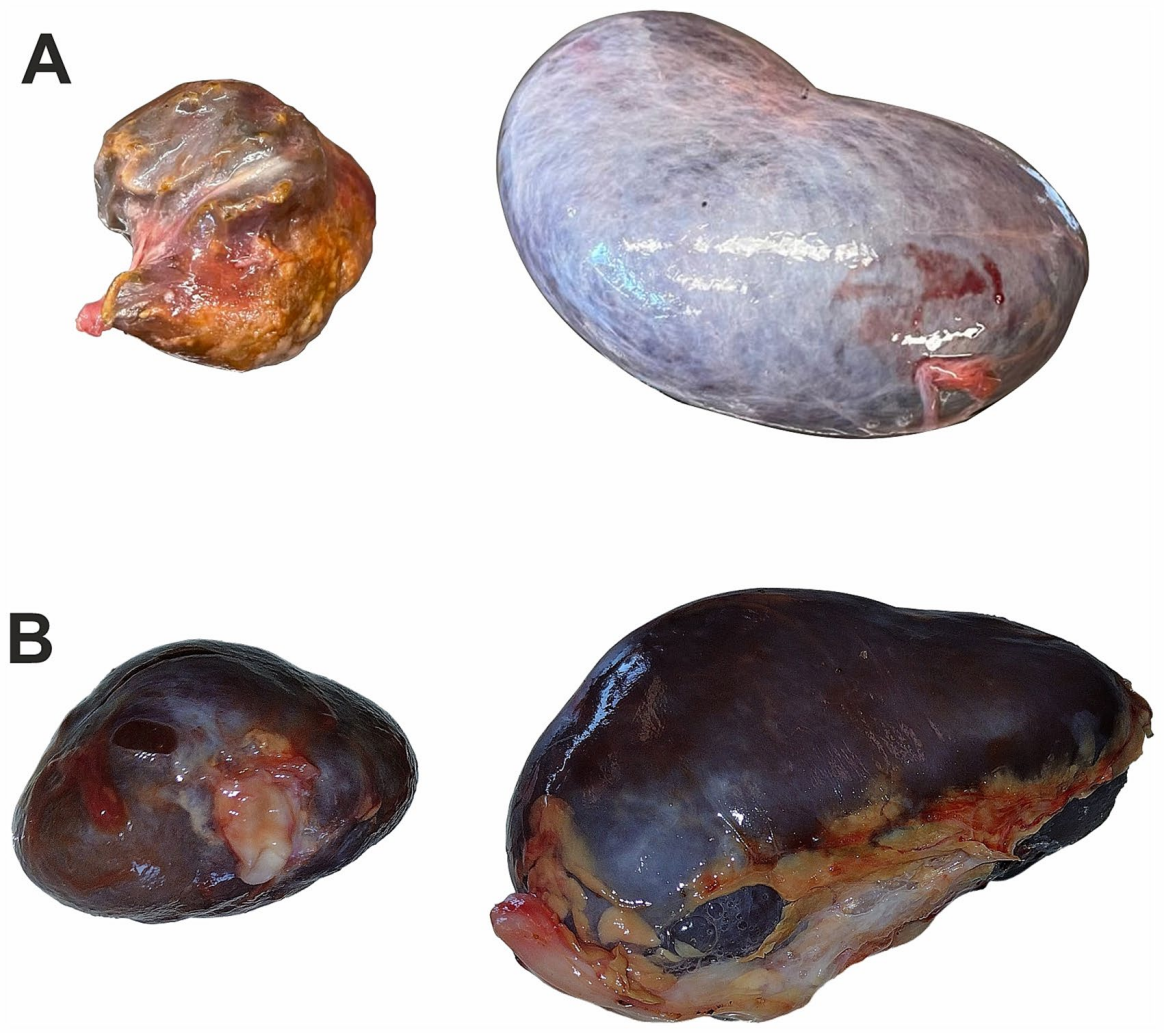
Kidney image from roe deer: **(A)** a kidney of roe deer in case 1 with a malformation (left) and a random example of the normal kidney (right), **(B)** a kidney of roe deer case 2 with a malformation (left) and a second normal-sized kidney (right); photos were taken at similar resolution and show differences in size.

Roe deer was hunted near Węgrów (52°23′3.10″N, 21°48′43.10″E), approximately 50 km northeast of Warsaw. The 5-year-old female was hunted during the winter of hunting season 2022/2023 (on 23 December 2022). The area is covered mainly with agricultural fields, small forest patches, and settlements. The smaller kidney from roe deer female was only 5 cm long and weighed approximately 20 g compared to 85.8 g of the larger kidney. On gross examination, the smaller kidney was shrunken with an uneven surface from cream-yellow to brown, and the capsule was hard to remove (Figure 1A).

**Figure 2:**
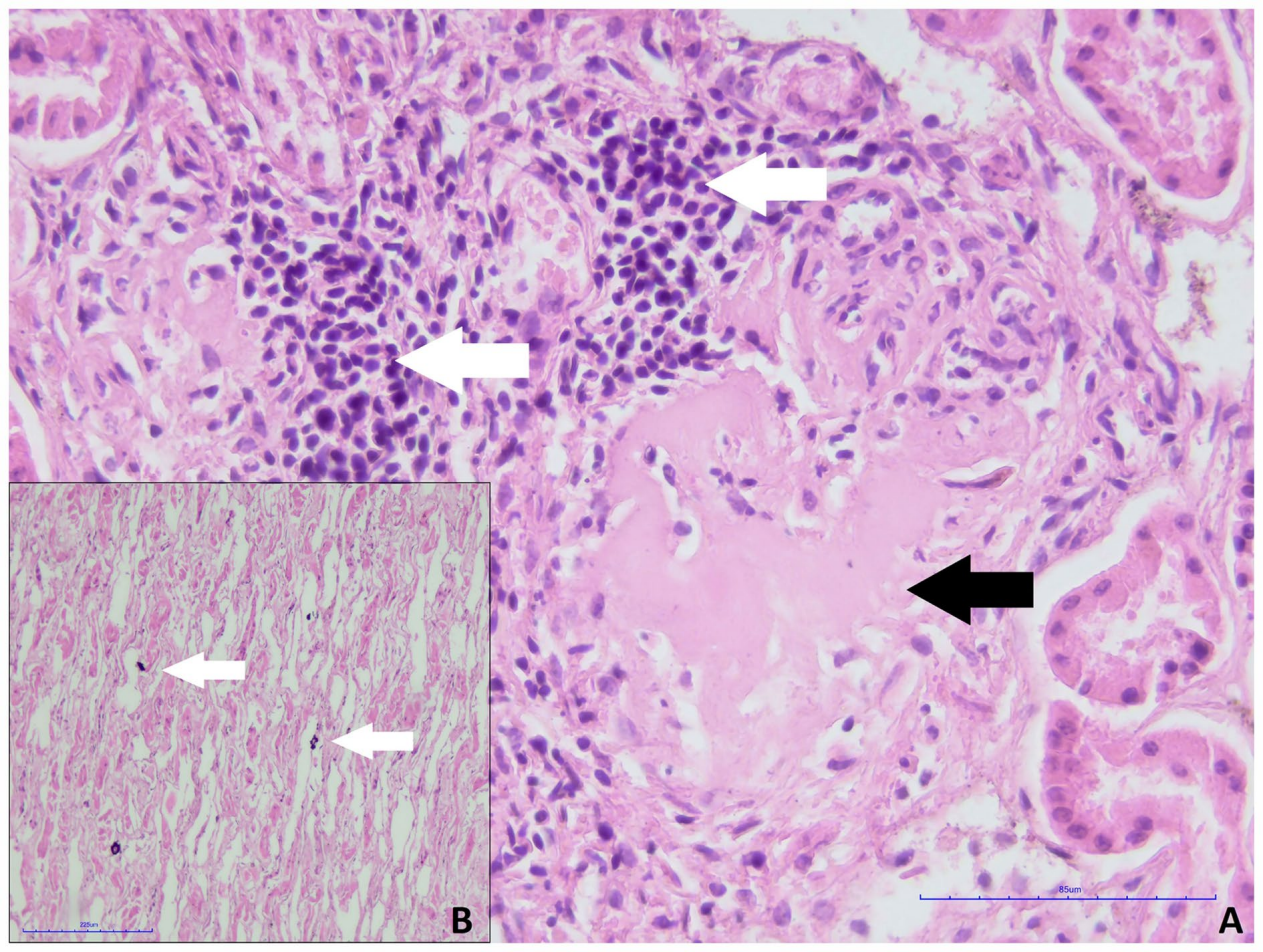
Smaller kidney of roe deer case 1. **(A)** Focal inflammatory infiltrate composed of lymphocytes and plasma cells (white arrows) and glomerular sclerosis (black arrow, H&E staining, magn. 400x). **(B)** Multifocal deposition of calcium salts within the renal medulla (white arrows, H&E staining, magn. 200x).

During the microscopic examination of the smaller kidney of roe deer, no prominent histopathological changes were found, and glomeruli were well developed. Only focally mild inflammatory infiltrate composed of mononuclear cells (lymphocytes, plasma cells) and glomerular sclerosis was present (Figure 2A). Multifocally small areas of calcification in the renal medulla were found (Figure 2B). No features of massive fibrosis, persistent metanephric duct, cartilage/osseous metaplasia, or other structures typical for renal dysplasia were present in the kidney. A diagnosis of renal hypoplasia was made concerning the size of the kidney, its macroscopic appearance, and histopathological picture. Unfortunately, no samples of the larger kidney were available due to damage to the organ before preparation for the histopathological examination.

## 3 Case 2

Roe deer was hunted near Rawa Mazowiecka (51°48′22.07″N 20° 4′29.36″E), approximately 75 km southwest of Warsaw. The 7-year-old male was hunted during the summer of hunting season 2024/2025 (on 25 August 2024). The area is covered mainly by agricultural fields dissected by settlements. The smaller kidney from roe deer male was 4.5 cm long and weighed 15.3 g in comparison to 7.5 cm and 55.6 g of the larger kidney. On gross examination, the smaller kidney was dark red in color with an uneven surface, and the capsule could be easily removed (Figure 1B).

**Figure 3:**
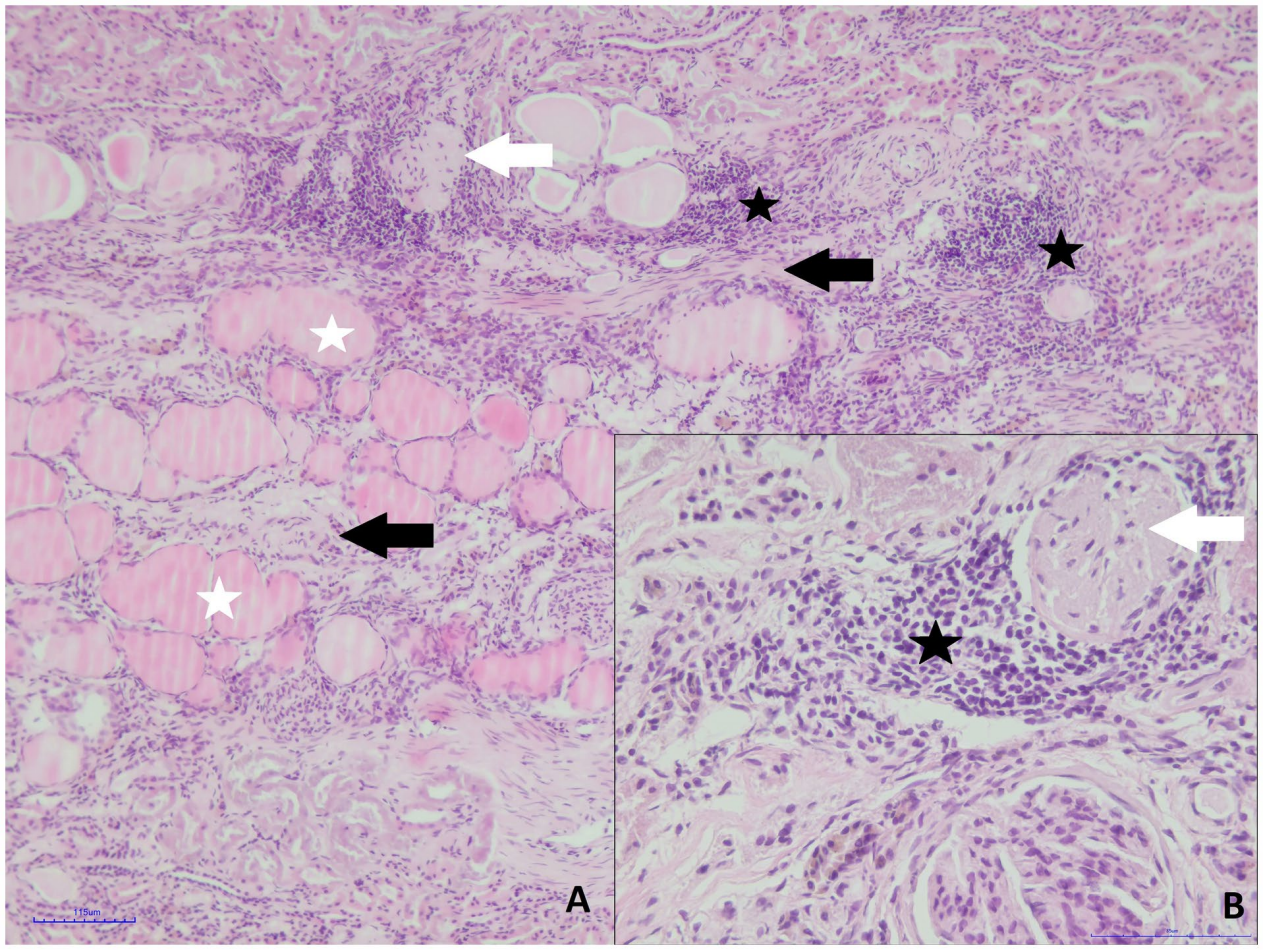
Roe deer case 2. **(A)** Massive mononuclear infiltrates (black asterisk), hyperplasia of the connective tissue (black arrows), glomerular sclerosis (white arrow) and hyaline casts in the tubular lumen (white asterisks, H&E staining, magn. 100x); **(B)** inflammatory infiltrate composed of small lymphocytes and plasma cells (asterisk) and glomerular sclerosis (white arrow, H&E staining, magn. 400x).

**Figure 4:**
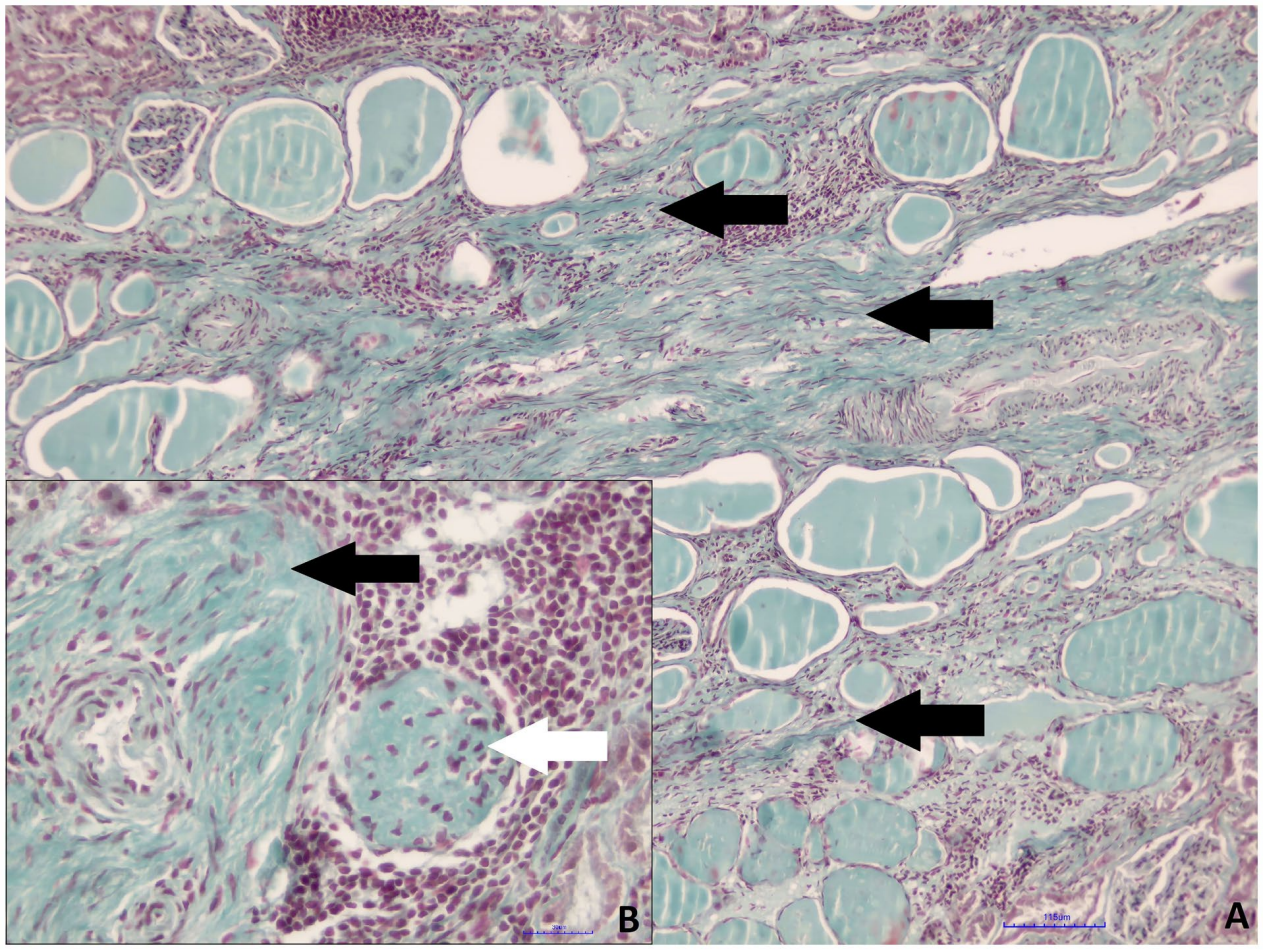
Roe deer case 2. **(A)** Hyperplasia of the interstitial connective tissue (black arrows, Masson’s trichrome staining, magn. 100x); **(B)** perivascular connective tissue hyperplasia (black arrow) and glomerular sclerosis (white arrow, Masson’s trichrome staining, magn. 400x).

Both kidneys of roe deer male were affected by similar pathological changes, although in the small kidney, they were significantly more prominent. Massive mononuclear infiltrates composed mainly of lymphocytes and plasma cells in the renal interstitium, with hyperplasia of the connective tissue and multifocal glomerular sclerosis, were found (Figures 3A,B). The presence of connective tissue was confirmed by Masson’s Trichrome staining (Figures 4A,B). Homogenous acidophilic masses corresponding to hyaline casts in the tubular lumen, indicating proteinuria (Figure 3A). Massive inflammatory infiltrates were also present in the area of the renal pelvis, where they were of a mixed nature, with an admixture of neutrophils. No calcification features were found in either H&E or Koss staining. Diagnosis of chronic interstitial nephritis was made. Because of the presence of inflammatory infiltrate in the area of the renal pelvis the spread of the process via the ascending route cannot be excluded. IgG and IgM Leptospira antibodies were not detected in tested samples.

## 4 Discussion

We described two renal malformations in a roe deer, resulting in a significant reduction in the size of the kidneys. Despite the similar macroscopic appearance, these lesions differ significantly in terms of etiopathology and microscopic appearance.

The reduction in kidney size resulting from its hypoplasia results from incomplete development of this organ in fetal life. It can occur both unilaterally and bilaterally. In the case of unilateral hypoplasia, compensatory hypertrophy of the other kidney is usually present (15). In the case of roe deer female (Case 1), the larger kidney weighed 85.8 g. In comparison, the average kidney weight in roe deer from both studied areas was 46.2 g (average of 331 kidneys, Klich, unpublished data), which indicates that compensatory hypertrophy was present. Previous studies have shown that maternal food restriction, low-protein diet, placental insufficiency, and maternal vitamins can cause congenital renal anomalies in the fetus (16). Both roe deer lived in the areas of large wind farms. Previous studies have shown that animals of this species may present increased physiological stress in response to large wind farms (17). One cannot recognize this infrastructure as the cause of this malformation. It should be, however, noted that the wind farm in Węgrów was established in 2015 and 2016, i.e., in 2022, it was functioning for 6 years. In case 1 female roe deer with hypoplasia, fetal development occurred during the wind farm’s establishment or the first year of its operation, which is the period of the most significant impact of this infrastructure on mammals [e.g., (18, 19)]. Conversely, the wind farm in Rawa Mazowiecka was established in 2014, i.e., in 2024, and has been functioning for 10 years. In case 2 – roe deer male (with chronic interstitial nephritis), the farm was established several years before the birth of this individual. Despite theoretical grounds for suspecting a negative impact of wind farms on health (20, 21), there is little research concerning animal health. Most studies include mammal frequency/density, behavior, and related issues [e.g., (22–25)]. However, some studies indicate increased stress in mammals in wind farms, although this effect was species-dependent [e.g., (17, 26)]. A negative impact on animal condition has been observed in pigs, i.e., lower muscle pH, heme iron, and *α*-linolenic acid in animals raised near wind turbines (27). Adverse health effects on animals may also be observed in other taxonomic groups; for example, domestic geese showed higher stress levels and lower activity levels when exposed to wind turbines (28). This issue requires further research in wildlife. Chronic interstitial nephritis, on the other hand, is usually of bacterial or viral origin. The most common etiological factors include leptospirosis, adenoviruses, lentiviruses, and herpesviruses (29). However, tests performed on roe deer case 2 samples did not confirm our leptospirosis assumptions. An increased content of heavy metals may also be a potential cause of inflammation in the kidney, and Poland is one of the countries with the highest emissions of heavy metals in Europe (30). An example is cadmium (Cd), which, once absorbed into the bloodstream, spreads throughout the body via circulation and accumulates in organs such as the kidneys (31). Cadmium exposure leads not only to disturbances in the function of renal tubules but also to general kidney dysfunction (32–34). In the natural environment, animals can be exposed to this element due to its use in agricultural and industrial production (35, 36) and its presence in sewage (37). The study areas from which the samples came are far from industry but are under intensive agriculture. Current data on cadmium concentration in roe deer in central Poland (where study areas are located) indicate relatively average levels but high levels in southern Poland (38). Elevated cadmium levels, however, were observed in other large herbivores in Poland, including central and eastern Poland (39–41). Other heavy metals can also cause neuropathy (42). Moreover, the trophic transfer of heavy metals has important implications not only for wildlife but also for human health (43). Therefore, further research is necessary to determine the possible content of heavy metals and their effect on the kidneys in the context of One Health (44). In particular, the roe deer is Poland’s most frequently harvested game animal, and its offal is used in the meat industry.

Typical changes in chronic interstitial nephritis include inflammatory infiltrates with a predominance of mononuclear cells, atrophic changes, fibrosis of the glomeruli sclerosis of varying degrees, and hyperplasia of fibrous connective tissue. These histopathological changes are not typical for renal hypoplasia. In the kidney of case 1, there was a single focal area of mild inflammatory infiltrate, probably reflecting a concomitant minor infection. Hyperplasia of connective tissue and secondary atrophy of renal structural components leads to its reduction in size. In case 2 of roe deer, inflammation was present in both kidneys, but greater intensity in one of them caused a significant size reduction compared to the other kidney. Because of the presence of inflammatory infiltrates in the renal pelvis, the ascending route of the process should be considered. In such cases, infections usually begin in the lower urinary or genital systems, spreading through the parenchyma of the kidney and becoming chronic (15, 29).

## Data Availability

The original contributions presented in the study are included in the article/supplementary material, further inquiries can be directed to the corresponding author.
